# lwreg: A Lightweight System for Chemical Registration and Data Storage

**DOI:** 10.1021/acs.jcim.4c01133

**Published:** 2024-08-08

**Authors:** Gregory A. Landrum, Jessica Braun, Paul Katzberger, Marc T. Lehner, Sereina Riniker

**Affiliations:** Department of Chemistry and Applied Biosciences, ETH Zurich, Vladimir-Prelog-Weg 2, 8093 Zurich, Switzerland

## Abstract

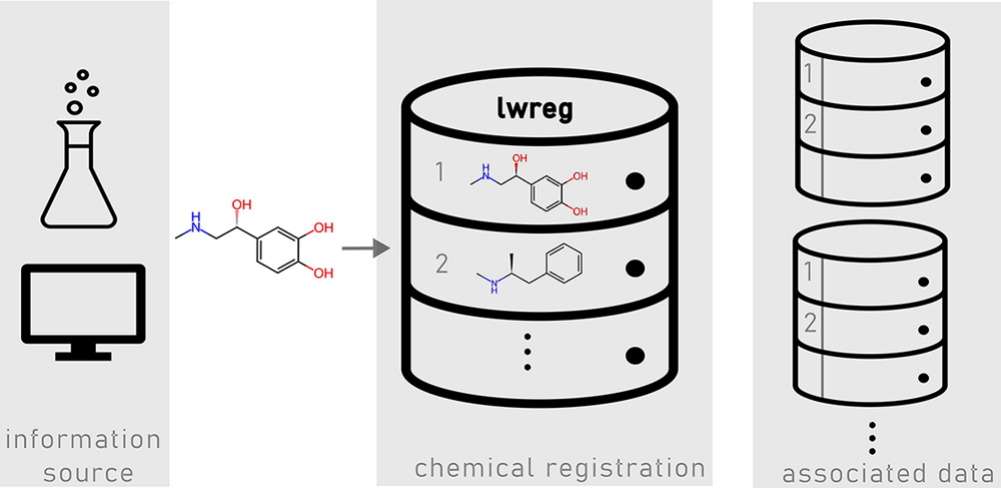

Here, we present lwreg, a lightweight, yet flexible chemical registration system supporting the capture of both two-dimensional molecular structures (topologies) and three-dimensional conformers. lwreg is open source, with a simple Python API, and is designed to be easily integrated into computational workflows. In addition to lwreg itself, we also introduce a straightforward schema for storing experimental data and metadata in the registration database. This direct connection between compound structural information and data generated using those structures creates a powerful tool for data analysis and experimental reproducibility. The software is available at and installable directly from https://github.com/rinikerlab/lightweight-registration.

## Introduction

For the purposes of this contribution, a chemical registration system is a database (or other software system) that stores chemical structures together with assigned identifiers. A minimal registration system needs to provide functionalities to check if a particular compound has already been registered and either return its associated identifier or add the new compound to the database. The registration system must also allow retrieval of a registered chemical structure via its identifier. The identifiers from registration systems are often used as keys in other databases (or tables withing the same database) to associate data with the structures. Chemical registration systems are familiar in chemistry and biology laboratories in industrial research environments, but are less common in academia and downright rare in computational laboratories in both industry and academia.

Some of this poor adoption of compound registration by computational groups is no doubt due to cultural/social factors, but there are some real technical and scientific challenges as well. On the technical side, most registration systems are applications with graphical user interfaces (GUIs), which are optimized for the manual registration of one compound (or at most a small number of compounds) at a time. These types of interfaces do not integrate well into the workflow of a computational scientist who may be working with large numbers of virtual molecules. For this, some kind of software library or application-programming interface (API) is required. On the scientific side, the biggest challenge with compound registration is that the answer to the question “are these two compounds the same?” is very context dependent. For example, at the simplest level, “standard” compound registration systems are inherently 2D and would recognize different conformers of the same molecule as being duplicates. These systems are not useful for someone who needs to track things like different docking poses or crystal structures of the same molecule. A more subtle problem is that many registration systems do some form of charge and/or tautomer standardization. This is again a problem if one needs to track the actual tautomer that was observed in an experiment or that was used as input to something like a molecular dynamics (MD) simulation.

Some possible use cases for the application of a chemical registration system alone or combined with a database for storing experimental results within a computational group include

- Tracking virtual compounds that have been considered and perhaps shared with experimental collaborators.
- Tracking the training and validation sets used for machine-learning models.
- Avoiding inadvertently duplicating expensive computations, like MD simulations or large quantum mechanics calculations, by tracking which compounds have already been run and where the results are stored.

Here, we introduce and describe lwreg, an open-source and highly flexible compound registration system, which is intended to be applicable across a broad range of computational workflows. The system is highly configurable, allows different definitions of chemical identity, and supports integration of experimental results. We focus exclusively on computational use cases here, however, lwreg could also be used in a “wet chemistry” setting. lwreg provides a simple, but flexible, Python API for registering compounds and querying the underlying database along with a command-line interface (CLI) allowing it to be integrated in scripting pipelines. Note that although it is possible to build a GUI intended for use by nonexpert users on top of the lwreg library, lwreg itself does not provide a GUI.

The rest of this contribution describes the design and implementation of lwreg itself, introduces how the context-dependent chemical identity is implemented and used, describes a simple approach for storing experimental results together with the compound registration data, and showcases a few of the ways that we have been using it in our research group.

## Methods

### Implementation and Interface

lwreg is implemented in pure Python with a very limited number of external dependencies other than the RDKit.^1^ The GitHub repository includes installation instructions for beginners along with an environment.yml, which makes it easy to try the lwreg tutorials using the conda package manager for Python. The package provides a simple CLI as well as a Python API.

The primary operations supported by lwreg are

- **register** – Attempts to register a new compound. If this is successful, a new registration ID (molregno) will be returned. There is also a bulk_register function for more efficiently registering more than one molecule at a time.
- **query** – Queries the registration database to determine whether or not a compound has already been registered and returns the corresponding molregno(s). The query command supports specification of which hash layers should be used in the comparison (see below for a discussion of the hash layers) and, as a result, may return multiple results.
- **retrieve** – Returns the registered molecule structure(s) for one or more molregnos. The retrieve function can optionally return either the structure that was submitted for registration (prestandardization) or the various hashes for a particular structure.

### Filtering and Standardizing Compounds

A common first step in chemical registration systems is to standardize molecules—removing fragments, transforming functional groups into a standard form, etc.—and then to check each molecule against a set of filters to ensure that they are compliant with the rules of the registration system. These filters may include things like flagging molecules that are badly drawn, include certain undesired elements or functional groups, or that have unspecified or incorrectly specified stereochemistry. The standardization rules and filters that are applied are generally specific to a particular company or institution. In order to support this important part of registration, lwreg provides a flexible and configurable interface for standardizing molecules before they are registered and for filtering out molecules that should not be added.

The built-in standardization operations currently include, among others, standard RDKit sanitization, fragment removal, and neutralization. The built-in filters include checks for overlapping atoms and molecules with SGroup data indicating that they are polymers. The full list is available in the GitHub repository. The current set is fairly minimal and we anticipate that more standardization and filtering options will be added as lwreg evolves. Adding new options is quite straighforward. It just requires implementing a new Python class that inherits from a class provided by lwreg and providing a name, some documentation, and a single function that returns a molecule (the input molecule for filters or the modified molecule for standardization operation) when successful or None on failure. It is possible to apply multiple standardization operations and filters in a user-specified order. The default standardization and filters for an lwreg database are stored as configuration information in the database itself and the particular operations that were applied for each molecule are stored with that molecule’s information in the database.

### Recognizing Duplicate Compounds

The heart of any chemical registration system is the method used to determine whether or not a given compound is already registered. Rather than building chemical querying into the underlying database, lwreg handles duplicate identification by computing a hash for each molecule in the database. To check whether or not a candidate molecule has already been registered, lwreg calculates a hash for the molecule and then checks whether or not that hash is already in the database. This test is done using simple string comparisons and is very fast in any modern database system. The method for computing a registration hash is discussed below.

### Registration Hash

The registration hashes, i.e., molecular hashes for registration, used by lwreg are generated by the RDKit. The RDKit’s RegistrationHash has previously been described in a brief presentation,^2^ but we give an overview here.

The hash layers provided by RegistrationHash are

1. FORMULA – The molecular formula
2. CANONICAL_SMILES – The molecule’s canonical CXSMILES. This includes information about stereochemistry (including enhanced stereochemistry)
3. TAUTOMER_HASH – The HetAtomTautomer hash (either v1 or v2) from MolHash. This includes information about stereochemistry (including enhanced stereochemistry)
4. NO_STEREO_SMILES – The canonical CXSMILES for the molecule without any information about stereochemistry
5. NO_STEREO_TAUTOMER_HASH – The HetAtomTautomer hash (either v1 or v2) for the molecule without any information about stereochemistry
6. SGROUP_DATA – A canonicalized form of some of the molecule’s SGroup data (if any). Which SGroup data fields are used can be configured by the user
7. ESCAPE – A free-text field allowing arbitrary information to be used as part of the molecule hash

**Figure 1:**
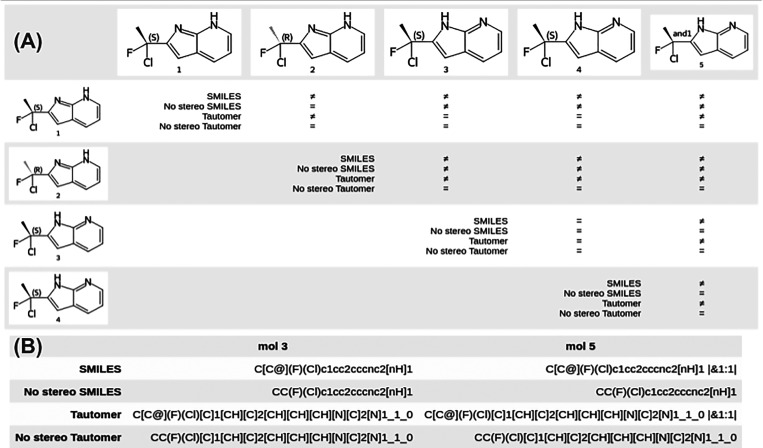
(A) Illustration of whether or not two molecules are considered to be the same by some of the layers in the registration hash. (B) Examples of the layers used to generate the registration hash for two of the two molecules.

Figure 1 shows the impact of the hash layers on whether or not two molecules are considered to be the same as well as the actual contents of the hash layers for two molecules.

The first five layers are generated using the RDKit’s implementation of the MolHash software from O’Boyle and Sayle.^3^ The layer containing the molecule’s SGroup data (if present) is handled by canonicalizing the data and then converting it to a simple JSON representation. The inclusion of the ESCAPE layer (i.e., a free-text field allowing arbitrary information to be incorporated into the registration hash) is inspired by the molecule key used as part of the Novartis small-molecule registration system.^4^

The RegistrationHash library generates the registration hash by computing an SHA1 hash from all of these layers using Python’s built-in hashlib library. lwreg uses this hash at registration time to determine whether or not a molecule has already been registered.

### Why Not InChI?

The IUPAC International Chemical Identifier^5^ (InChI), either in its standard form or one of the nonstandard forms, provides a hash that could be used for molecular identity checking. InChI is also composed of a variety of layers, which can be carefully extracted and stored to provide some of the layers mentioned above. However, we opted not to use InChI in lwreg so that we have full control over both the molecular standardization and details of the hashing. Standardization is an integral part of the InChI algorithm and cannot be disabled.^6^

### Querying Using Hash Layers

The default behavior of a query in lwreg is to standardize the query molecule, calculate the default registration hash, and then use that to query the table with molecule hashes. This returns the molregno of the matching entry or None if no match is found. It is also possible to specify which layers of the registration hash should be used. Depending on which layers are selected, this allows querying for, for example, registered molecules that are tautomers or stereoisomers of the query. When the system is in registerConformers mode and the query molecule has a conformer, the conformer hash is calculated for the query molecule and used to query the conformer_hash table. Matches are returned as (molregno, conf_id) tuples.

A final option for the query function, available only when the system is in registerConformers mode, is to retrieve all conformer IDs for a list of query molregnos.

Chemical queries such as substructure or similarity queries are not possible with the base functionality of lwreg. These require a connection to a chemically aware search function like that provided by the RDKit PostgreSQL cartridge, see below.

### Retrieving Structures

The retrieve function allows structural information about one or more molecules to be retrieved from the database. Structures are returned as v3000 mol blocks. When in registerConformers mode, the registered conformers will be returned if (molregno, conf_id) tuples are provided.

Instead of the registered structure, it is also possible to retrieve either the “as registered” form (this is exactly what was provided at registration time) or all of the mol hashes for a particular molregno.

### Storing Conformers

We use a simple hashing scheme to quickly recognize whether or not a particular conformer has already been seen in the database. The algorithm used for hashing a conformer is

1. Convert the *x*, *y*, *z* coordinates of each atom into strings by rounding each coordinate to a particular number of digits after the decimal (default: 3).
2. Describe the position of each atom as a string by concatenating the strings of the individual coordinates together with commas.
3. Sort the string representations of the atom positions lexicographically.
4. Construct the final hash by concatenating the sorted string representations together with semicolons and generating the SHA256 hash of the result.

Note that this simple scheme is independent of the atom ordering but it is neither translationally nor rotationally invariant. This is essential to allow the system to be used for storing prealigned conformers (e.g., for storing docking poses). If the user desires translational and/or rotational invariance, they should either standardize the orientation of conformers themselves before registering them or use the CanonicalizeOrientation step in the standardization pipeline to automate that process.

### Storing Experimental Data

Though lwreg itself is focused on registering chemical structures, we find it very useful to employ lwreg together with some additional database tables to store data from experiments, including computational experiments, carried out using the registered molecules. Given the lack of flexible lightweight systems for storing experimental data, and the tremendous benefits it brings to have experimental results easily accessible and queryable together with the molecules used in the experiments, we briefly describe our approach here. We also provide tutorials (Jupyter notebooks) demonstrating how experimental data can be captured and used in the lwreg GitHub repository.

**Figure 2:**
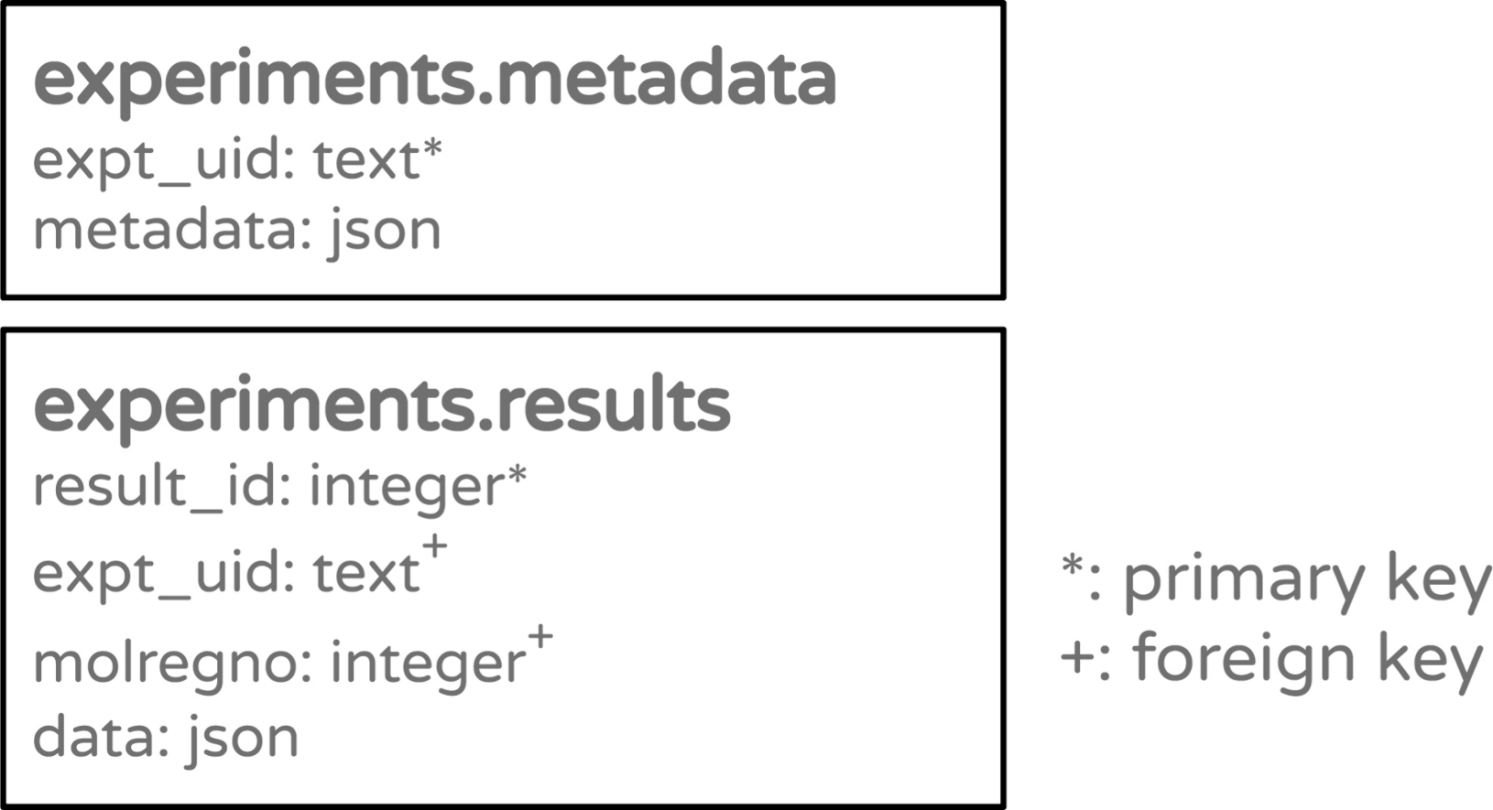
A minimal database schema for storing experimental data. As discussed in the text, though this simple schema can store the results from almost any experiment type, it is often useful to use a schema with explicit columns for at least some of the result types.

Our general approach is to store experimental metadata—the information required to reproduce the experiment—and data—the results from the experiments—in separate tables. When storing different types of experiments in one database, we use one table per experiment type. We use universally unique identifiers (UUIDs) as keys to identify the individual experiments and to link the experimental data to the associated metadata. This is shown schematically in Figure 2 and illustrated with examples in the tutorial Jupyter notebooks.

We store the experimental results in tables using a combination of standard numeric and text columns and JSON (or JSONB) columns. The standard columns allow compact storage and very fast querying/retrieval of data, while the JSON/JSONB columns allow complete flexibility in how the data is stored. A few concrete examples are provided in the Results and Discussion section below.

### Adding Chemical Search: Integration With the RDKit Cartridges

lwreg does not directly support performing chemical searches like similarity or substructure searches. However, users wishing to do chemical searches on their lwreg tables can install either the RDKit’s PostgreSQL cartridge or Riccardo Vianello’s chemicalite cartridge for sqlite.^7^ Because this is not part of lwreg itself and installing and configuring either of these database add-ons can be nontrivial and specific to a particular database deployment, we only highlight the possibility for this integration here and will not go into detail.

## Results and Discussion

We have done several benchmarking exercises with lwreg and applied it in multiple projects in our lab. This usage has helped us to refine the API and available options. Here, a selection of our use cases is presented.

All of the performance tests reported here were run by executing the lwreg commands on a two-year old workstation with a 3.8 GHz Intel Xeon W-1270P CPU and 64 GB of RAM running Ubuntu 22.04. The database was a PostgresSQL v14 installation running on an eight-year old workstation with a 3.5 GHz Intel Xeon E3–1240 CPU and 32GB of RAM running Ubuntu 22.04. The default Ubuntu PostgreSQL configuration was used without further performance tuning.

### Registering All Compounds From ChEMBL33

The first use case we explored is a simple one: Initialize a new lwreg database with a large collection of molecules. In this case, we loaded all of the compounds from the ChEMBL33^8^ SD file and we used basic RDKit sanitization for the standardization.

The final database contains 2.3 million molecules. The full registration data for the molecules occupies about 7.4 GB of disk space: ∼1 GB for the molecule hashes, ∼3.0 GB for the original mol blocks, and ∼3.4 GB for the mol blocks table. Total runtime for the registration was ∼17.5 h (26 ms per molecule on average). Query performance was tested using 1000 random mol blocks from the ChEMBL33 data set and the lwreg query command. The median query time was 5.6 ms with 80% of the queries completing in less than 16 ms. The maximum query time was 274 ms.

### Storing DASH Tree Conformers

In order to test the performance of lwreg when registering conformers as well as compounds, we considered the collection of compounds and conformers used to parametrize the DASH tree in ref (9). The database contains 365’419 molecules and 1’027’559 conformers. Conformers were stored without hydrogen atoms. The conformer table occupies 3.5 GB of disk space, while the registration data for the molecules themselves occupies about 1.3 GB of disk space. We did three performance tests with this database. Determining whether or not a particular conformer was present in the database and, if so, retrieving the associated molregno and conformer ID took 220 ms for 100 molecules (2.2 ms per molecule on average). Determining whether or not a particular molecule was present in the database and retrieving the associated molregno took 250 ms for 100 molecules (2.5 ms per molecule on average, which is essentially the same timing as the equivalent query for the ChEMBL database discussed above). Finally, registering one new conformer for each of 100 molecules took 1.7 s for the 100 molecules (17 ms per molecule on average).

### Storing Molecular Data Sets for Bioactivity Prediction and Associated Machine-Learning Results

For the next use case, we were interested in tracking how well various machine-learning algorithms and descriptors perform at building predictive models on the SIMPD bioactivity data sets.^10^ Here, we initialized the database by registering all of the compounds from each of the SIMPD data sets. During this setup process, the lwreg database was extended to include a schema that contained a registry of data sets. The registration system was configured to use the ChargeParent standardization, automatically capturing only the largest organic fragment from each compound and neutralizing it. Metadata about the machine-learning runs and the results of the experiments on the individual data sets was stored in another set of tables in the database. An example of how to set up and use these tables is present in the tutorial folder of the lwreg repository.

### Tracking Conformer Generation Results

**Figure 3:**
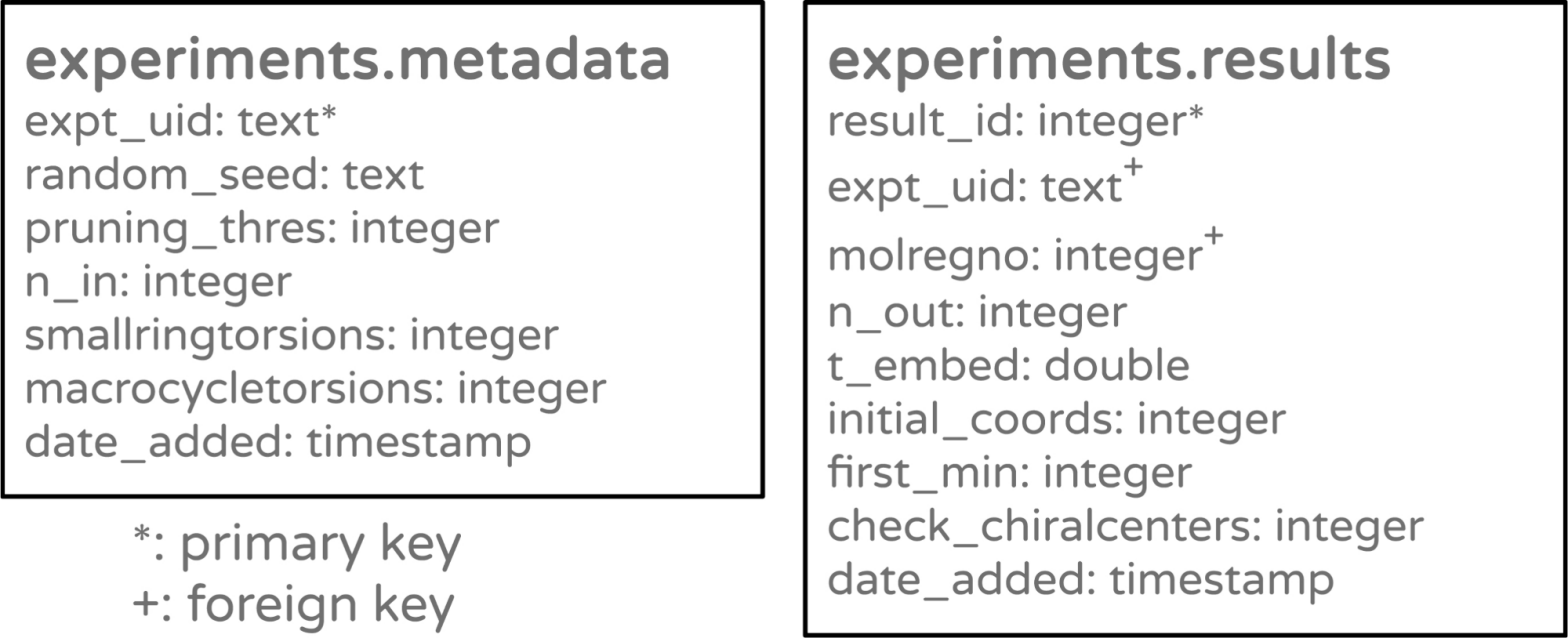
Database schema for storing results from the flexibility experiments.

Our last example use case for tracking experimental results using lwreg is from an active project where we are looking at understanding molecular flexibility based on the results from, and failures of, the RDKit’s conformer generation algorithm. In contrast to the previous use cases, here we have fully modeled both the metadata and data from the experiments and thus are not using JSON columns in either the metadata or data tables. This approach of using columns for everything we want to capture has the advantages of being more explicit and higher performance while also requiring less storage space—the last two are factors that might be relevant for very large databases—at the cost of being marginally less flexible. If we want to capture additional data or metadata for a few experiments, we would need to add additional columns to the tables. The database schema is shown in Figure 3.

## Conclusions

lwreg provides a flexible and easy to setup tool for registering the compounds used in a laboratory. The use of a layered hash for duplicate detection allows the system to store multiple variations (tautomers, stereoisomers, conformers, etc.) of the “same” compound, while still allowing very fast queries to retrieve related structures. We have also shown how the compound registration schema provided by lwreg can be augmented by additional tables, which are used to store experimental results, resulting in a lightweight system that can track metadata describing experiments (including computational experiments), the data from those experiments, and the compounds that were used in the experiments.

As lwreg provides a simple Python API, it can be easily integrated into other computational workflows either on its own or together with a set of supplemental tables to track experimental results. The lack of a GUI means that lwreg itself is probably not directly useful as a compound registration system for scientists who are not regularly working with Python, however, we think that it provides a solid and extensible back-end upon which such a system could be built.

We have found both the compound-storage and data-storage pieces of lwreg to be very useful in our own research and hope other researchers do as well. We are happy to answer questions about the tools in the “Discussions” section of the GitHub repository as well as to get bug reports and/or feature requests in the issue tracker.

## Data Availability

lwreg and the sample Jupyter notebooks are available under the MIT open-source license in our GitHub repository: https://github.com/rinikerlab/lightweight-registration.
